# Chocolate and risk of chronic disease: a systematic review and dose-response meta-analysis

**DOI:** 10.1007/s00394-019-01914-9

**Published:** 2019-02-25

**Authors:** Jakub Morze, Carolina Schwedhelm, Aleksander Bencic, Georg Hoffmann, Heiner Boeing, Katarzyna Przybylowicz, Lukas Schwingshackl

**Affiliations:** 1 Department of Human Nutrition, University of Warmia and Mazury, ul. Sloneczna 45f, 10-718 Olsztyn, Poland; 2 Department of Epidemiology, German Institute of Human Nutrition Potsdam-Rehbruecke (DIfE), Arthur-Scheunert-Allee 114-116, 14558 Nuthetal, Germany; 3 NutriAct-Competence Cluster Nutrition Research Berlin-Potsdam, 14458 Nuthetal, Germany; 4 Department of Nutritional Sciences, University of Vienna, Althanstraße 14, UZA II, 1090 Vienna, Austria; 5 Institute for Evidence in Medicine, Medical Center, University of Freiburg, Breisacher Straße 153, 79110 Freiburg, Germany

**Keywords:** Chocolate, Meta-analysis, Dose-response, Credibility of evidence, Chronic disease

## Abstract

**Purpose:**

Evidence for the association between chocolate intake and risk of chronic diseases is inconclusive. Therefore, we aimed to synthesize and evaluate the credibility of evidence on the dose-response association between chocolate consumption with risk of all-cause mortality, coronary heart disease (CHD), stroke, heart failure (HF), type 2 diabetes (T2D), colorectal cancer (CRC), and hypertension.

**Methods:**

Prospective studies were searched until July 2018 in PubMed, Embase, and Web of Science. Random-effects meta-analyses comparing highest versus lowest intake categories, linear, and non-linear dose-response analyses were conducted. The credibility of evidence was evaluated with the NutriGrade scoring-system.

**Results:**

Overall, 27 investigations were identified (*n* = 2 for all-cause mortality, *n* = 9 for CHD, *n* = 8 for stroke, *n* = 6 for HF, *n* = 6 for T2D, *n* = 2 for hypertension and CRC, respectively). No associations with HF (RR 0.99, 95% CI 0.94, 1.04) and T2D (RR 0.94, 95% CI 0.88, 1.01) per each 10 g/day increase in chocolate intake were observed in the linear dose-response meta-analyses. However, a small inverse association for each 10 g/daily increase could be shown for the risk of CHD (RR 0.96, 95% CI 0.93, 0.99), and stroke (RR 0.90, 95% CI 0.82, 0.98). The credibility of evidence was rated either very low (all-cause mortality, HF, T2D, CRC or hypertension) or low (CHD, stroke).

**Conclusion:**

Chocolate consumption is not related to risk for several chronic diseases, but could have a small inverse association with CHD and stroke. Our findings are limited by very low or low credibility of evidence, highlighting important uncertainty for chocolate–disease associations.

**Electronic supplementary material:**

The online version of this article (10.1007/s00394-019-01914-9) contains supplementary material, which is available to authorized users.

## Introduction

Cocoa, mostly consumed as chocolate in Western countries, is rich in various bioactive compounds such as flavanols including catechins, epicatechin, and proanthocyanidins as well theobromine [1]. The flavanols of cocoa have been found to exert beneficial effects on endothelial function, platelet aggregation, insulin sensitivity, oxidative damage, and inflammation, all of which play a key role in the pathogenesis of major non-communicable diseases (NCD) including cardiovascular diseases (CVD), type 2 diabetes (T2D), and cancer [2].

Diet is recognized as a modifiable risk factor for NCD and a change in dietary behaviour is a cornerstone for disease prevention [3]. However, the inclusion of chocolate as a part of dietary recommendations for NCD prevention remains controversial. Nonetheless, the European Food Safety Authority (EFSA) argued that a daily consumption of 10 g of high-flavanol dark chocolate might improve vasodilatation without disturbing a balanced diet [4]. On the other hand, many food grouping systems classify chocolate together with confectionary and sweets, and dietary recommendations favour lower intakes of this food group due to its high content of fat and added sugar [5, 6].

Previous meta-analyses of prospective studies have shown that chocolate intake is associated with decreased risk of coronary heart disease (CHD), T2D, heart failure (HF), and stroke [7–9]. Moreover, dose-response meta-analyses revealed a non-linear association with the highest protective effect of 2–3 servings of chocolate per week for T2D and HF risk as well as a small decrease of risk with higher intakes for CHD and stroke [8, 9]. However, these meta-analytical findings were limited due to the fact that the credibility of evidence was not assessed, which is highlighted by a recently published umbrella review [10]. To assess the association between different food groups and chronic diseases as well as mortality, we have already performed a number of meta-analyses focusing on the strength and dose specificity of these associations [11–16]. This included calculations of both linear as well as non-linear dose-response relationships. To complement and to be consistent with these analyses, the present study aimed to summarize the evidence on the relationship between chocolate consumption and risk of either all-cause mortality, CHD, stroke, hypertension, CRC, or T2D. In addition, the NutriGrade scoring system was implemented to evaluate the credibility of the evidence for the derived correlations.

## Methods

The review was registered in PROSPERO International Prospective Register of Systematic Reviews (ID: CRD42016037069). The methodological procedure for conducting this review was based on a previously published protocol [11], that was already implemented in a number of reviews. This meta-analysis followed the guidelines for reporting Meta-analyses of Observational Studies in Epidemiology (MOOSE) [12].

### Search strategy

Literature search was performed until July 2018 to identify relevant articles in the electronic databases PubMed, Embase (Ovid), and Web of Science. Full search strategies for all three sources are listed in ESM Material 1. Citation lists from retrieved articles, systematic reviews, and meta-analyses were searched for additional studies. Moreover, we searched Google Scholar for articles citing or associated with included articles, which could meet the inclusion criteria. Two authors (JM, LS) conducted the literature search, while any uncertainty was resolved by consensus of third reviewer (HB).

### Study selection

Studies were included in the systematic review and meta-analysis if they (1) were cohorts, case–cohorts, case–control nested in cohort studies, as well as follow-ups of randomized controlled trials; (2) investigated the association between chocolate consumption with risk of all-cause mortality, CHD, stroke, heart failure, CRC, T2D, or hypertension in adults (aged ≥ 18 years). Corresponding chronic diseases were defined using information from previously published meta-analyses [13–19].

Two authors (JM, LS) screened and extracted the following data independently: first author’s name, year of publication, country, study name, study design, baseline age of participants, sex, sample size, number of cases, dietary assessment method, outcome, outcome assessment method, quantity of chocolate intake, multivariable effect estimate with corresponding 95% confidence intervals (CIs), and covariates. If only separate estimates for male and female participants were reported in a study, the risk ratios (RRs) were pooled using a fixed-effect model.

### Statistical analysis

For high versus low and dose-response comparisons, we applied a random-effects model to derive pooled RRs and 95% CIs [20], summarizing the associations between chocolate consumption and risk of all-cause mortality, CHD, stroke, HF, CRC, T2D, and hypertension. Using an inverse variance method, the standard error (SE) for the log-transformed RR was calculated and interpreted as an estimated variance of log-transformed RR to weight each study [20]. For purposes of this meta-analysis, we assumed that all measures are RRs. A method described by Greenland and Longnecker was applied for the linear dose-response meta-analysis [21, 22]. Information on RRs with 95% CI, number of cases and person-years or non-cases, was required for at least three quantitative exposure categories for the implementation of this method. Dose-response meta-analyses were conducted if ≥ 3 studies were available for each corresponding outcome. If a study already reported an estimated linear dose-response trend with 95% CI or SE, it was directly included in our analyses.

If studies reported only the total number of cases or person-years and the exposure was defined in categories, we obtained the number of person-years or cases in each category by dividing the total number of person-years/cases per number of reported categories, as it was previously described [11]. The median and mean intake of chocolate, respectively, was assigned by quantile to the corresponding risk estimate. If studies included intakes only as a range by quantile, the midpoint was calculated. For open-ended intake ranges, we assumed that the width was the same as the contiguous category. If the exposure was expressed per given unit of energy intake, we used the provided mean energy intake to rescale it.

The dose-response was expressed as 10 gram/d of chocolate. If a study did not provide information on the amount of chocolate per serving, 28.5 g/d (1 oz.) were used as serving size [23].

If more than three categories of exposure were provided by a study, restricted cubic splines were calculated to explore possible non-linear associations. Three fixed knots were used through the total range of the reported intake at 10%, 50%, and 90% and combined using multivariate meta-analysis [24].

To explore heterogeneity between studies, the Cochran *Q* test and the *I*^2^ statistic were used. A value for the *I*^2^ statistic greater than 50% was considered as potentially important statistical heterogeneity [25]. Subgroup analyses were performed, if more than five studies were available for an outcome in the linear dose-response analysis. Subgroup analyses included stratification for sex (male/female/both), length of follow-up (mean or median ≥ 10 years/< 10 years), geographic location (by continent), number of cases (≥ 1000/< 1000), validation of dietary assessment method (validated/not validated), adjustment for dietary energy intake (adjusted/not adjusted) and type of chocolate intake used for risk estimation (pure/all-source).

According to the Cochrane Handbook, we explored potential small-study effects such as publication bias using Egger´s test and funnel plots, if 10 or more studies were available [26]. Statistical analyses were conducted using Stata version/SE 14.2 software (StataCorp, College Station, TX) and Review Manager 5.3 (Nordic Cochrane Centre, Copenhagen).

### Credibility of the evidence

To evaluate the credibility of evidence for the association between chocolate consumption and risk of all-cause mortality, CHD, stroke, heart failure, T2D, CRC, and hypertension, a recently developed NutriGrade scoring system was implemented (max 10 points). This tool is based on the following criteria for prospective studies: (1) risk of bias, study quality, study limitations (up to 2 points), (2) precision (up to 1 point), (3) heterogeneity (up to 1 point), (4) directness (up to 1 point), (5) publication bias (up to 1 point), (6) funding bias (up to 1 point), (7) effect size (up to 2 points), and (8) dose-response (up to 1 point) [27]. The following categorization of the calculated score is recommended to interpret the credibility of evidence: high (≥ 8 points), moderate (6 to < 8 points), low (4 to < 6 points), and very low (0 to < 4 points).

## Results

Out of the 287 records identified by the literature search, 47 full-text articles were assessed in detail (ESM Material 2, ESM Ref), and 27 were included in the meta-analysis as they reported on chocolate consumption and at least one of 7 diseases (ESM Fig. 1).

**Fig. 1:**
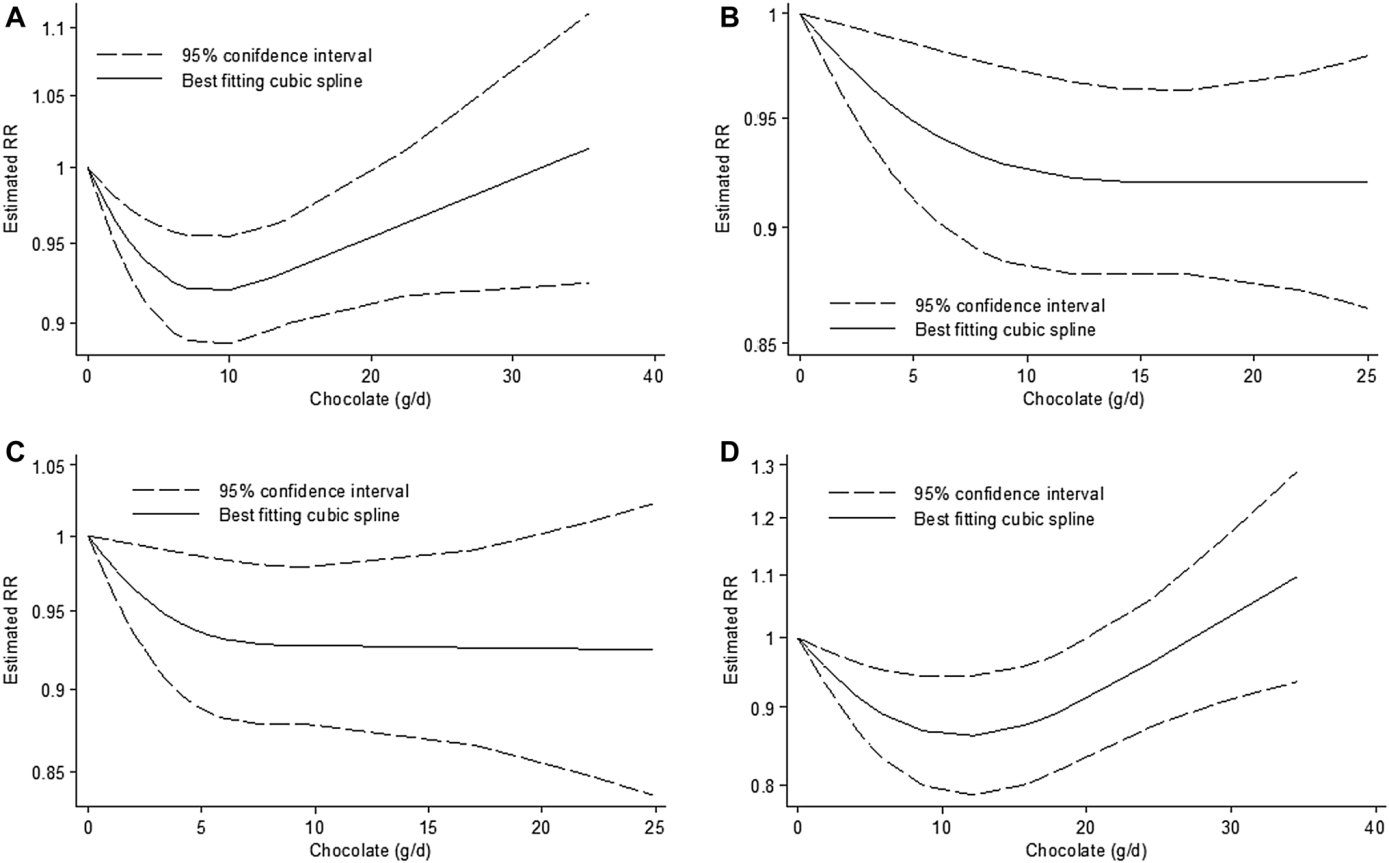
Non-linear dose-response relation between daily intake of chocolate and (**a**) risk of type 2 diabetes mellitus (*p*_non-linearity_ = 0.002; *n* = 6 studies), (**b**) coronary heart disease (*p*_non-linearity_ = 0.07; *n* = 8 studies), (**c**) stroke (*p*_non-linearity_ = 0.06; *n* = 7 studies) and (**d**) heart failure (*p*_non-linearity_ < 0.001; *n* = 5 studies)

Two prospective observational studies were included for all-cause mortality [28, 29], 9 reports (10 studies) for CHD [28, 30–37], 8 studies for stroke [28, 30, 31, 33, 36, 38–40], 6 studies for heart failure [28, 35, 41–44], 6 studies for T2D [23, 45–49], and 2 studies for CRC [50, 51], and hypertension [52, 53], respectively (ESM Table 1).

### All-cause mortality

Two studies with 11,596 death cases were included in the highest versus lowest intake category meta-analysis (overall intake range 0–17 g/d). No association between all-cause mortality and chocolate intake was observed (RR 0.98; 95% CI 0.93, 1.03, *I*^2^ = 0%, *p*_heterogeneity_ = 0.44) when comparing extreme categories (ESM Fig. 2). Due to the limited availability of data, it was not possible to conduct any further meta-analyses.

### Coronary heart disease, stroke, and heart failure

Nine prospective observational studies with 21,294 CHD cases, eight studies with 11,949 stroke cases, and 6 studies with 4606 HF cases, were included in the highest versus lowest intake category meta-analysis (range of intake 0–46.1 g/d). Comparing categories of highest versus lowest intake of chocolate intake, we observed no association with risk of CHD (RR 0.92; 95% CI 0.85, 1.00, *I*^2^ = 51%, *p*_heterogeneity_ = 0.04) (ESM Fig. 3) and HF (RR 0.87; 95% CI 0.71, 1.06, *I*^2^ = 53%, *p*_heterogeneity_ = 0.06) (ESM Fig. 4), whereas an inverse association was observed for risk of stroke (RR 0.86; 95% CI 0.76, 0.96, *I*^2^ = 61%, *p*_heterogeneity_ = 0.01) (ESM Fig. 5).

Similarly, an increase in chocolate intake by 10 g per day was not associated with risk of HF (RR 0.99, 95% CI 0.94, 1.04, *I*^2^ = 29%, *p*_heterogeneity_ = 0.23, *n* = 5) (ESM Fig. 6), but was inversely related with risk of CHD (RR 0.96; 95% CI 0.93, 0.99, *I*^2^ = 29%, *p*_heterogeneity_ = 0.21, *n* = 8) (ESM Fig. 7) and stroke (RR 0.90, 95% CI 0.82, 0.98, *I*^2^ = 59%, *p*_heterogeneity_ = 0.02, *n* = 7) (ESM Fig. 8).

In additional analyses stratified by sex, follow-up duration, number of cases, dietary assessment method, chocolate type, and energy adjustment, no statistically significant subgroup differences were observed (ESM Tables 2 and 3). However, in the subgroup analysis considering geographic location, CHD and stroke showed an inverse association in European studies, but not in US studies.

The non-linear dose-response analyses showed a borderline significance for CHD (*p*_non-linearity_*p* = 0.07, *n* = 8 studies) and stroke (*p*_non-linearity_*p* = 0.06, *n* = 7 studies) and significance for HF (*p*_non-linearity_*p* < 0.001, *n* = 5 studies) **(**Fig. 1**)**. The risk of CHD and stroke decreased by approximately 7–8% with increasing intake of chocolate up to ~ 20 g/d, with no further risk decreasing association above this intake level. The risk of HF decreased by approximately 14% with increasing intake of chocolate up to ~ 12 g/d, and a trend for a risk increasing association was observed with intakes > 35 g/d **(**Fig. 1**)**.

### Type 2 diabetes

Six studies with 21,758 incident T2D cases were included in the meta-analysis comparing extreme intake categories (range of intake 0–35.4 g/d). We observed an inverse association between risk of T2D and chocolate consumption (RR 0.87; 95% CI 0.79, 0.97, *I*^2^ = 60%, *p*_heterogeneity_ = 0.03) comparing extreme categories (ESM Fig. 9). A linear increase in chocolate intake by 10 g per day was not associated with risk of T2D (RR 0.94; 95% CI 0.88, 1.01, *I*^2^ = 75%, *p*_heterogeneity_ = 0.001, *n* = 6) (ESM Fig. 10).

In additional analyses, no subgroup differences for chocolate consumption were observed for sex, length of follow-up, geographic location, number of cases, dietary assessment method, chocolate type, or energy adjustment (ESM Table 4).

There was a non-linear dose-response trend (*p*_non-linearity_ = 0.002, *n* = 6 studies). The risk of T2D decreased by approximately 8% with increasing intake of chocolate up to ~ 10 g/d, and a trend for a risk increasing association was observed with intakes > 30 g/d **(**Fig. 1**)**.

### Colorectal cancer

Only 2 studies with 1368 incident colorectal cancer cases were identified (range of intake 0–10.7 g/d). No association between colorectal cancer and chocolate intake was observed (RR 1.05; 95% CI 0.75, 1.47, *I*^2^ = 32%, *p*_heterogeneity_ = 0.23) when comparing extreme categories (ESM Fig. 11). Due to the limited availability of data it was not possible to conduct linear and non-linear dose-response meta-analyses.

### Hypertension

Only 2 studies with 9530 incident hypertension cases were identified (range of intake 0–18 g/d). No association between hypertension and chocolate intake was observed (RR 0.97; 95% CI 0.91, 1.04, *I*^2^ = 0%, *p*_heterogeneity_ = 0.59) when comparing extreme categories (ESM Fig. 12). Due to the limited availability of data, it was not possible to conduct linear and non-linear dose-response meta-analyses.

### Credibility of evidence

Overall, the credibility of evidence for the association between chocolate intake and risk of all-cause-mortality, HF, T2D, CRC, and hypertension was rated “very low”, whereas the credibility of evidence for the association between chocolate consumption and risk of CHD and stroke was rated as “low” (ESM Table 5). Overall, there is very low or low confidence in the effect estimate, and the evidence is (very) limited and uncertain.

## Discussion

The present systematic review and meta-analysis examined the association between chocolate intake and risk of all-cause mortality, CHD, CRC, HF, hypertension, stroke, and T2D using data from 27 prospective studies, including more than 1 million participants. For stroke, we found a high versus low risk gradient coupled with a linear dose-response relation, and with some indication of non-linearity of the relation. For CHD, we found no significant high versus low gradient, but a linear dose-response and also an indication of non-linearity of the relation. For HF and T2D, we found also a high versus low risk gradient but coupled with a non-linear dose-response relation. Interestingly, the non-linear analyses indicated an increased risk with consumption above 20–30 g/d for HF. For all-cause mortality, CRC, and hypertension we found no associations. However, the credibility of evidence assessed with the NutriGrade tool did not exceeded the grade low for CHD and stroke, and very low for the other endpoints.

Previous meta-analyses focused predominantly on CVD [7, 8, 54–57] and found an inverse relation. Interestingly, one meta-analysis reported an inverse non-linear association between chocolate and risk of overall CVD, with the peak of a protective association at 45 g of chocolate per week [7], which is equivalent of 6–7 g/d. However, results of the meta-analyses on CHD varied with the included endpoints. If only myocardial infarction (MI) was analyzed, a recently published meta-analysis suggested a 16% lower risk in high versus low comparison, as well as a 1.9% decrease per 20 g/week of chocolate in a dose-response manner [7]. If CHD was analyzed, high chocolate consumption was associated with a 10% lower risk of CHD in high versus low comparisons [8]. Additionally, an inverse non-linear association when increasing the dose of chocolate consumption was identified [8]. In our meta-analysis chocolate intake was only borderline inversely associated with CHD risk in the non-linear dose-response comparison. The shape of the non-linear risk association indicated no further risk reduction when consuming more than 20 g/d. Concerning high versus low intake analyses, the interpretation of findings across all studies might be limited due to a large range of intakes used in the included studies. Our dose-response results confirm previous findings about chocolate lowering the risk of CHD, but with no evidence of profitable high consumption effects [8]. Similar observations might be found in the case of stroke. Our results are in line with earlier summaries showing that risk of stroke decreases according to both high versus low and linear dose-response comparisons [7]. Moreover, a non-linear dose-response analysis revealed that risk of stroke was not lowered when chocolate intake exceeded 20 g/d. Regarding HF, our findings are in line with previous reports showing no association in high versus low comparison [7, 41]. In the non-linear dose-response analysis, we found a significant non-linear pattern with a peak risk reduction at 12 g/d intake and a slight trend of increasing risk when amount consumed exceeds 35 g/d, which is confirmed by another meta-analysis [9].

Regarding T2D, we found a 13% lower risk when comparing high and low chocolate consumption and a clear non-linear association suggesting lowest risk when consuming 10 g/d of chocolate and no association when the amount consumed exceeds 30 g/d. Similarly to the findings of HF, the credibility of evidence for T2D is very low.

Our meta-analysis is the first summarizing the evidence between chocolate consumption and risk of hypertension as well as CRC. In both cases no associations were observed. Results on risk of all-cause mortality were in line with previous data, showing no association with chocolate intake [56].

There are several mechanisms which are proposed to explain the link between chocolate and health effects. Polyphenols including flavanols such as catechin, epicatechin and polymeric proanthocyanidins are main bioactive substances found in cocoa extracts [1]. Cocoa contains a higher amount of flavanols compared to other sources such as red wine, apples or tea, and therefore it has been considered as a potential target for dietary interventions [54]. Protective effects of cocoa flavanols include antioxidants, free-radical scavenging, antiplatelet and anti-inflammatory actions as well as improvement of endothelial function, via increased bioavailability of nitric oxide [2]. Reduction of local oxidative stress and lipid peroxidation, as well as platelet activation, in turn, may prevent the development of atherosclerosis [2]. A recent meta-analysis of randomized controlled trials indicated that cocoa flavanol intake may decrease serum triglycerides and C-reactive protein, improve insulin sensitivity and increase high-density lipoprotein [58]. According to the recent Cochrane review, there is moderate credibility of evidence that consumption of high-flavanol chocolate may cause a small reduction of blood pressure, which is one of the major risk factors of stroke [59]. In our meta-analysis we observed no association between chocolate intake and risk of hypertension, however, the estimate was based only on two studies and had been addressing risk of hypertension among non-hypertensives and not the reduction of blood pressure among hypertensives. Apart of polyphenols, cocoa is also a rich dietary source of theobromine. Several studies suggested that theobromine may enhance flavanol-caused decrease of blood pressure, pulse velocity, increased flow-mediated dilatation and high-density lipoprotein [60, 61].

Chocolate is an energy-dense food and contains a relatively high amount of saturated fat and added sugar [62]. An increased energy density of a meal might be associated with lower quality of diet, and a higher BMI and waist circumference, which are directly related to NCD [63]. In prospective studies, higher chocolate intake was associated with greater weight gain [64]. Inverse associations between chocolate and BMI found in cross-sectional studies might be explained by a change of dietary habits in participants with chronic diseases [65]. This is a potential source of bias for studies included in this meta-analysis as they reported risk in relation to chocolate intake assessed at baseline.

Due to the high energy density of chocolate, adjustment for energy intake is a reasonable approach to exclude potential cofounding [66]. However, subgroup analysis with stratification for energy adjustment showed no difference between risk estimates. Energy misreporting may have a greater influence on the diet-disease relationship than energy intake itself. In the cross-sectional study by Gottschald and colleagues, adjustment for energy misreporting reversed initial inverse or null association between confectionery products and BMI, as well as cardiometabolic risk factors [67]. Estimates in our meta-analysis could be affected as none of the included studies accounted for energy misreporting, which in particular is often present in overweight and obese people [68].

There is evidence that the link between health outcomes and chocolate consumption might differ depending on the type of chocolate consumed [2]. Dark chocolate contains more cocoa and has a higher amount of flavanols than milk chocolate and therefore may have increased protective effects. Both in Europe and the US, milk chocolate consumption is higher than that of dark chocolate [2, 28]. Unfortunately, food frequency questionnaires used in all included studies did not distinguish between dark and milk chocolate consumption. Therefore, a potential protective effect of dark chocolate might be attenuated by that of other types of chocolate. Moreover, in this meta-analysis, there was no significant difference in subgroup analysis stratified for chocolate type. However, in the plain chocolate studies, there was a decrease of CHD and stroke risk that was not present anymore in chocolate from all-source studies. Hence, identification of patterns of chocolate intake might be the key to understand the relationship between chocolate and disease.

## Strengths and limitations

Several limitations should be considered when interpreting the results of the present meta-analysis. Most of the included studies assumed a constant intake of chocolate using intake assessed at baseline. None of the identified studies distinguished between intakes of dark and milk chocolate. Moreover, some studies estimated chocolate intake including chocolate sweets, snacks, milk or general confectionery [23, 30, 31, 33, 46, 47, 49]; this might limit the interpretation of these results due to cofounding of fat and added sugar intake. For all-cause mortality and hypertension, only a limited number of studies (less than three) was available, therefore we could not conduct dose-response and subgroup comparisons.

Strengths of this meta-analysis are the inclusion of prospective studies only, usage of multiple meta-analytical comparisons to investigate high versus low, linear and non-linear dose-response relationships. Furthermore, we included conference abstracts and letters to the editor which published results from big and recognized cohort studies, not included in previous reviews. Additionally, we assessed credibility of evidence using the NutriGrade tool, which was rarely implemented in previous reviews and only for high versus low comparisons.

## Conclusions

Chocolate consumption is not related to risk of several chronic diseases, but could reduce risk of CHD and stroke. However, this finding is limited by low trust into such a conclusion, highlighting the uncertainty of health consequences of chocolate intake. Any discussion about the inclusion of small amounts of dark chocolate in food-based dietary guidelines need to consider this uncertainty. The presence of several methodological issues such as insufficient information on energy misreporting and type of chocolate should be taken into account in further prospective studies.

## Electronic supplementary material

Below is the link to the electronic supplementary material.

Supplementary material 1 (PDF 521 KB)
