# Supplementary material for: Chocolate and risk of chronic disease: a systematic review and dose-response meta-analysis
Source: Eur J Nutr. 2019 Feb 25;59(1):389–97. doi: 10.1007/s00394-019-01914-9 (PMC7000512; doi:10.1007/s00394-019-01914-9)
Supplement: Supplementary file 1 — Supplementary material 1 (PDF 521 KB) [file 394_2019_1914_MOESM1_ESM.pdf]

Chocolate and risk of chronic disease: a systematic review and dose-response meta-analysis

Jakub Morze[1], Carolina Schwedhelm[2,3], Aleksander Bencic[2], Georg Hoffmann[4], Heiner Boeing[2], Katarzyna Przybylowicz[1], Lukas Schwingshackl[2,3,5]

[1]Department of Human Nutrition, University of Warmia and Mazury, ul. Sloneczna 45f, 10-718 Olsztyn, Poland

[2]Department of Epidemiology, German Institute of Human Nutrition Potsdam-Rehbruecke (DIfE), Arthur-Scheunert-Allee 114-116, 14558 Nuthetal, Germany

[3]NutriAct-Competence Cluster Nutrition Research Berlin-Potsdam, 14458 Nuthetal, Germany

[4]Department of Nutritional Sciences, University of Vienna, Althanstraße 14, UZA II, 1090 Vienna, Austria

[5]Institute for Evidence in Medicine, Medical Center, University of Freiburg, Faculty of Medicine, University of Freiburg, Freiburg, Germany.

Corresponding author: Lukas Schwingshackl, PhD

Breisacher Straße 153, 79110 Freiburg, Germany

M: schwingshackl@ifem.uni-freiburg.de

T: +49 (0)761 203-96867

ELECTRONIC SUPPLEMENTARY MATERIAL

Abbreviations:

95%CI – 95% Confidence Interval;

ARIC – Atherosclerosis Risk in Communities Cohorts;

CHD – Coronary Heart Disease;

COSM – Cohort of Swedish Men;

COOW – Prospective Study of Clinical Outcomes in Older Women;

E3N – Etude Epidémiologique auprès de femmes de la Mutuelle Générale de l'Education Nationale;

EPIC – European Prospective Investigation into Cancer and Nutrition;

FHS – Framingham Heart Study;

HR – Hazard Ratio;

IWHS – Iowa Women Health Study;

JPHC – Japan Public Health Center-based Prospective Study;

LWCS – Leisure World Cohort Study;

M – Men;

MEC – Multiethnic Cohort Study;

MSLS – Maine-Syracuse Longitudinal Study;

MVP – Million Veteran Program;

NA – Not applicable;

NHS – Nurses' Health Study;

OR – Odds Ratio;

PC – Prospective Cohort;

PHS- Physicians' Health Study;

RCT – Randomized Controlled Trial;

RR – Risk Ratio;

SHEEP – Stockholm Heart Epidemiology Program;

SMC – Swedish Mammography Cohort;

SUN – Seguimiento Universidad de Navarra Study;

T2D – Type 2 diabetes mellitus

TS – Takayama Study;

W – Women;

WHI – Women's Health Initiative;

ZES – Zutphen Elderly Study;

ESM Material 1:

PubMed search strategy:

#1 chocolate[tiab] OR cocoa[tiab]

#2 mortality[tiab] OR death[tiab]

#3 cardiovascular[tiab] OR coronary[tiab] OR stroke[tiab] OR vascular[tiab] OR myocardial infarction[tiab] OR heart failure[tiab]

#4 colon[tiab] OR rectum[tiab] OR rectal[tiab] OR colorectal[tiab] OR colorectum[tiab] OR bowel[tiab]

#5 diabetes[tiab]

#6 hypertension[tiab] OR hypertensive[tiab] OR systolic[tiab] OR diastolic[tiab] OR blood pressure[tiab]

#7 prospective[tiab] OR cohort[tiab] OR longitudinal[tiab] OR follow-up[tiab] OR case-cohort[tiab] OR nested case-control[tiab]

#8 (#1 AND (#2 OR #3 OR #4 OR #5 OR #6) AND #7)

Embase search strategy:

#1 chocolate.ti,ab. OR cocoa.ti,ab.

#2 mortality.ti,ab. OR death.ti,ab.

#3 cardiovascular.ti,ab. OR coronary.ti,ab. OR stroke.ti,ab. OR vascular.ti,ab. OR myocardial infraction.ti,ab. OR heart failure.ti,ab.

#4 colon.ti,ab. OR rectum.ti,ab. OR rectal.ti,ab. OR colorectal.ti,ab. OR colorectum.ti,ab. OR bowel.ti,ab.

#5 diabetes.ti,ab.

#6 hypertension.ti,ab. OR hypertensive.ti,ab. OR systolic.ti,ab. OR diastolic.ti,ab. OR blood pressure.ti,ab.

#7 prospective.ti,ab. OR cohort.ti,ab. OR longitudinal.ti,ab. OR follow-up.ti,ab. OR case-cohort.ti,ab. OR nested case-control.ti,ab.

#8 (#1 AND (#2 OR #3 OR #4 OR #5 OR #6) AND #7)

Web of Science search strategy:

#1 TS=chocolate OR TS=cocoa

#2 TS=mortality OR TS=death

#3 TS=cardiovascular OR TS=coronary OR TS=stroke OR TS=vascular OR TS=myocardial infraction OR TS=heart failure

#4 TS=colon OR TS=rectum OR TS=rectal OR TS=colorectal OR TS=colorectum OR TS= bowel

#5 TS=diabetes

#6 TS=hypertension OR TS=hypertensive OR TS=systolic OR TS=diastolic OR TS=blood pressure

#7 TS=prospective OR TS=cohort OR TS=longitudinal OR TS=follow-up OR TS=case-cohort OR TS=nested case-control

#8 (#1 AND (#2 OR #3 OR #4 OR #5 OR #6) AND #7)

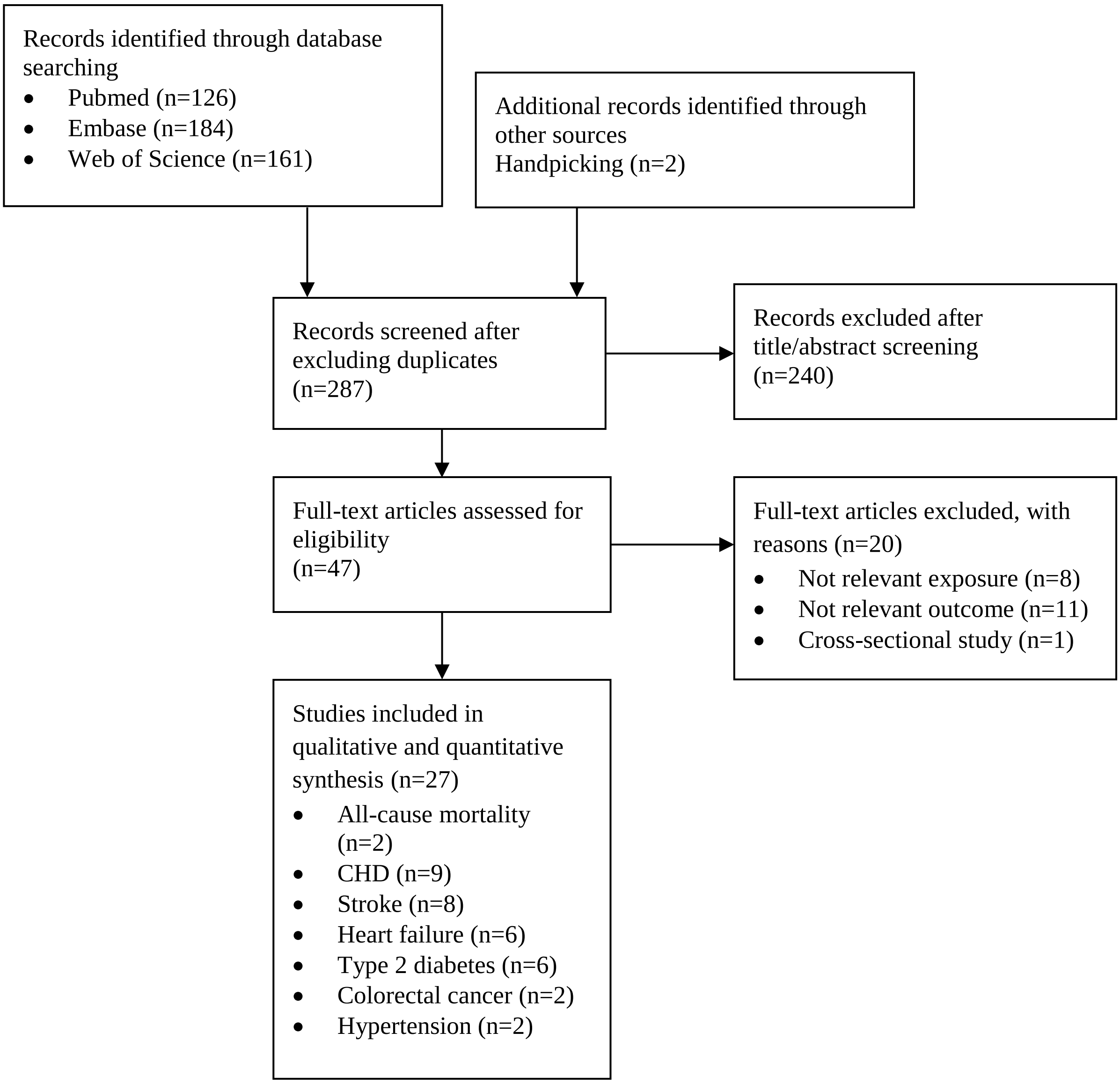

ESM Fig 1: Flow diagram illustrating identification and study selection process

ESM Material 2: Full-text articles with reason for exclusion

References	Reason for exclusion
[1-8]	Exposure not relevant
[9-19]	Outcome not relevant
[20]	Cross-sectional study

ESM Table 1: General study characteristics of the included studies investigating the association between chocolate intake and risk of all-cause mortality, coronary heart disease, stroke, heart failure, type 2 diabetes, and hypertension.

Author & Year	Study name & Country	Study Design	Age at entry	Sex	Sample size	Total cases	Dietary assessment	Outcome	Outcome assessment	Exposure	Adjustment factors	Follow up years	Consumption frequency or amount	RR/HR/OR (95% CI)
Alonso et. al. (2005)	SUN (Spain)	PC	35.8	M&W	5880	180	Validated FFQ	Hypertension	Self-reported	Chocolate (All-source)	Age, sex, BMI, physical activity, alcohol consumption, sodium intake, total energy intake, smoking, hypercholesterolemia, low-fat dairy consumption	2	g/d<br>0<br>2<br>4<br>6<br>18	 1.00<br>0.60 (0.40, 1.10)<br>0.90 (0.50, 1.50)<br>1.40 (0.90, 2.40)<br>1.10 (0.70, 1.80)
Buijsse et. al. (2010)	EPIC-Potsdam (Germany)	PC	35-65	M&W	19357	166	Validated FFQ	Myocardial infarction	Self-reported (verified by medical records)/ Death certificate	Chocolate (All-source)	Age, sex, alcohol intake, employment status, BMI, waist circumference, smoking status, occupational physical activity, sports, cycling, education, total energy intake, energy-adjusted intakes of fruit, vegetables, red meat, processed meat, dairy, coffee, tea, and cereal fibres, prevalence of diabetes	8.1	g/d<br>1.7<br>1.9<br>3.3<br>7.5	 1.00<br>0.65 (0.40, 1.05)<br>1.02 (0.65, 1.60)<br>0.73 (0.47, 1.15)
						136		Stroke					g/d<br>1.7<br>1.9<br>3.3<br>7.5	 1.00<br>0.79 (0.48, 1.29)<br>0.80 (0.48, 1.35)<br>0.52 (0.30, 0.89)
Crichton et. al. (2017)	MSLS (USA)	PC	24-96	M&W	590	30	Validated FFQ	T2D	Medical examination	Chocolate (Pure)	Age, education, sex, ethnicity, total cholesterol, obesity, hypertension, CRP, physical activity, total grains, coffee, red wine	4.7	servings/wk<br><1<br>1<br>>1	 1.91 (1.03; 3.55)<br>1.27 (0.64; 2.52)<br>1.00
Dong et. al. (2017)	JPHC (Japan)	PC	44-76	M&W	84597	M: 2025	Validated FFQ	Stroke	Medical records	Chocolate (Pure)	Age, area, BMI, smoking, sports, occupation, medication use for hypertension and hypercholesterolemia, intakes of alcohol, total energy, green tea,	12.9	g/wk<br>0<br>5.8<br>11.6<br>37.5	 1.00<br>0.98 (0.86, 1.13)<br>1.08 (0.96, 1.22)<br>0.94 (0.80, 1.10)

						F: 1533					coffee, fish/seafood, meat, fruits, soy foods, vegetable		g/wk<br>0<br>5.8<br>11.6<br>37.5	 1.00<br>0.90 (0.78, 1.04)<br>0.97 (0.84, 1.11)<br>0.84 (0.71, 0.99)
Greenberg et. al. (2015)	ARIC (USA)	PC	45-64	M&W	7802	861	FFQ	T2D	Medical Examination	Chocolate (All-source)	Age, race, sex, physical activity, smoking status, Keys Index of Dietary Quality, family history of diabetes, dietary energy intake, alcohol intake, educational level, occupational level,	13.3	g/d<br>0<br>2.8<br>14.2<br>35.4	 1.00<br>0.87 (0.75, 1.02)<br>0.66 (0.53, 0.82)<br>0.82 (0.62, 1.10)
Greenberg et. al. (2017)	WHI (USA)	PC	50-79	W	92678	10804	Validated FFQ	T2D	Self-reported (validated)	Chocolate (All-source)	Age, race/ethnic status, WHI Study arm, family history of diabetes, smoking status, total recreational energy expended in MET-h per week, coffee in cups per day, non-chocolate energy intake, Alternative Modified Health Eating Index, education, family income, physical functional ability, emotional well being	13.1	g/d<br>0<br>0.8<br>2<br>5.6<br>22.1	 1.00<br>0.97 (0.92, 1.04)<br>0.92 (0.87, 0.98)<br>0.93 (0.88, 0.98)<br>0.98 (0.92, 1.04)
Greenberg et. al. (2018)	WHI (USA)	PC	50-79	W	83310	3246	Validated FFQ	CHD	Medical records/ Death certificates	Chocolate (All-source)	Age, race/ethnicity, WHI study arm, Alternative Health Eating Index , nonchocolate daily energy intake, physical activity, smoking status, alcohol intake, educational level, income status, family history of CVD	13.4	g/d<br>0<br>0.9<br>2<br>5.7<br>22.5	 1.00<br>1.07 (0.96, 1.19)<br>1.06 (0.96, 1.18)<br>0.98 (0.89, 1.08)<br>1.03 (0.92, 1.15)
						2624		Stroke					g/d<br>0<br>0.9<br>2<br>5.7<br>22.5	 1.00<br>1.07 (0.95, 1.21)<br>1.04 (0.92, 1.17)<br>0.97 (0.87, 1.09)<br>1.09 (0.96, 1.23)
Ho et. al. (2018)	MVP (USA)	PC	64	M&W	148465	9040	FFQ	CHD	Medical records	Chocolate (Pure)	Age, sex, race, lifestyle factors	2.6	g/d<br>0<br>1<br>4<br>12<br>24.4	 1.00<br>0.93 (0.86, 1.12)<br>0.91 (0.82, 1.00)<br>0.91 (0.82, 1.00)<br>0.89 (0.80, 0.99)

Hu et. al. (2000)	NHS (USA)	PC	34-59	W	80082	939	Validated FFQ	CHD	Medical records/ Self-reported/ Death certificates	Chocolate (Pure)	Age, time period, BMI, cigarette smoking, menopausal status, parental history of myocardial infarction before age 60 y, vitamin E supplement use, alcohol consumption, history of hypertension, aspirin use, vigorous exercise, total energy intake	14	g/d<br>0<br>0.5<br>4<br>14	 1.00<br>1.15 (0.96, 1.37)<br>1.08 (0.88, 1.32)<br>1.11 (0.92, 1.34)
Janszky et. al. (2009)	SHEEP (Sweden)	PC	45-70	M&W	1169	210	Standardized questionnaire	All-cause mortality	Death certificates/ Medical records	Chocolate (Pure)	Age, gender, smoking, obesity, physical inactivity, alcohol consumption, filtered coffee consumption, educational attainment, sweet score	8.7	g/d<br>0<br>0.9<br>4.4<br>17	 1.00<br>0.89 (0.56, 1.42)<br>0.96 (0.63, 1.48)<br>0.94 (0.58, 1.53)
						250		Recurrent myocardial infarction					g/d<br>0<br>0.9<br>4.4<br>17	 1.00<br>0.95 (0.61, 1.49)<br>1.02 (0.68, 1.55)<br>0.86 (0.54, 1.37)
						279		Congestive heart failure					g/d<br>0<br>0.9<br>4.4<br>17	 1.00<br>0.82 (0.56, 1.19)<br>0.68 (0.47, 0.97)<br>0.78 (0.52, 1.16)
						111		Stroke					g/d<br>0<br>0.9<br>4.4<br>17	 1.00<br>0.67 (0.36, 1.23)<br>0.54 (0.30, 0.96)<br>0.62 (0.33, 1.16)
Kwok et. al. (2015)	EPIC-Norfolk (UK)	PC	59	M&W	16162	1754	Validated FFQ	CHD	Medical records/ Death certificates	Chocolate (All-source)	Age, sex, smoking, physical activity, energy intake, alcohol consumption, diabetes, BMI, systolic blood pressure, LDL cholesterol, HDL cholesterol, CRP	11.3	g/d<br>0<br>1.1<br>1.6<br>9.2<br>24.9	 1.00<br>1.00 (0.87, 1.14)<br>0.98 (0.84, 1.13)<br>0.87 (0.75, 1.01)<br>0.82 (0.70, 0.97)
						648		Stroke					g/d<br>0<br>1.1<br>1.6<br>9.2<br>24.9	 1.00<br>0.95 (0.76, 1.18)<br>0.87 (0.67, 1.11)<br>0.94 (0.74, 1.18)<br>0.80 (0.62, 1.05)

Kwok<br>et. al. (2016)	EPIC-Norfolk (UK)	PC	58	M&W	20922	1101	Validated FFQ	Heart failure	Medical records/ Death certificates	Chocolate (All-source)	Age, sex, education, BMI, social class, physical activity, smoking status, dietary energy, alcohol consumption, myocardial infarction, diabetes, arrhythmia, systolic blood pressure, cholesterol level, heart rate	12.5	g/d<br>0<br>0.8<br>4.1<br>8.7<br>24.9	 1.00<br>0.92 (0.77, 1.11)<br>0.96 (0.80, 1.15)<br>0.89 (0.74, 1.06)<br>0.87 (0.71, 1.06)
Lajous<br>et. al. (2016)	E3N (France)	PC	45-58	W	40574	9350	Validated FFQ	Hypertension	Self-reported/ Medical records	Chocolate (Pure)	Age, energy, education, history of hypertension, diabetes, hypercholesterolemia, BMI, smoking, physical activity metabolic equivalents, hormone therapy	13.8	Q1<br>Q5	1.00<br>0.97 (0.91, 1.03)
Larsson<br>et. al. (2011)	SMC (Sweden)	PC	49-83	W	33372	1548	Validated FFQ	Stroke	Medical records/ Death certificates	Chocolate (Pure)	Age, education, smoking status, BMI, total physical activity, aspirin use, self-reported history of hypertension, diagnosis of atrial fibrillation, family history of myocardial infarction before 60 years of age, intake of total energy alcohol, coffee, tea, fresh read meat, fish, fruits, and vegetables	10.4	g/d<br>1.3<br>2.0<br>4.1<br>9.5	 1.00<br>1.04 (0.86, 1.25)<br>0.94 (0.82, 1.08)<br>0.80 (0.66, 0.99)
Larsson<br>et. al. (2012)	COSM (Sweden)	PC	45-79	M	37103	1995	Validated FFQ	Stroke	Medical records/ Death certificates	Chocolate (Pure)	Age, education,. smoking status, pack-years of smoking, BMI, total physical activity, aspirin use, history of hypertension, atrial fibrillation, family history of myocardial infarction, intakes of total energy, alcohol, coffee, tea, fresh read meat, fish, fruits, and vegetables	10.2	g/d<br>0<br>1.8<br>5.5<br>9	 1.00<br>0.94 (0.84, 1.05)<br>0.95 (0.83, 1.08)<br>0.83 (0.70, 0.99)
Larsson<br>et. al. (2016)	COSM & SMC (Sweden)	PC	45-83	M&W	67640	4417	Validated FFQ	Myocardial infarction	Medical records/ Death certificates	Chocolate (Pure)	Age, sex, education, family history of MI before 60 years of age, aspirin, walking/bicycling, intakes of total energy, alcohol, processed meat, fruits and vegetables, BMI, history and diagnosis of diabetes, hypertension , hypercholesterolemia	13	g/d<br>0<br>1.9<br>6<br>14	 1.00<br>0.91 (0.84, 0.99)<br>0.89 (0.81, 0.97)<br>0.87 (0.77, 0.98)
Lewis<br>et. al. (2010)	COOW (USA)	RCT follow-up	≥70	W	1216	153	Validated FFQ	Ischemic heart disease	Medical records/ Death	Chocolate (Pure)	Age, BMI, socioeconomic status, energy intake at baseline	9.5	serving/wk<br><1<br>≥1	 1.00<br>0.65 (0.46, 0.94)

						53		Heart failure	certificates				serving/wk<br><1<br>≥1	 1.00<br>0.41 (0.22, 0.76)
Makarem<br>et. al. (2018)	FHS<br>(USA)	PC	26-84	M&W	3184	68	Validated FFQ	Colorectal cancer	Medical records (verified by pathology report)/Death certificates	Chocolate (All-source)	Age, sex, smoking, alcohol, energy, fiber intake and red and processed meat intake. BMI, WC, chronic diseases (CVD and diabetes), education, physical activity, antioxidant use, percentage energy from fat, fruit and vegetable intake	22	serving/wk<br><0.5<br>0.5-1.9<br>>1.9	 1.00<br>1.08 (0.62, 1.90)<br>0.74 (0.37, 1.46)
Maskarinec<br>et. al. (2018)	MEC<br>(USA)	PC	45-75	M&W	151691	8487	Validated FFQ	T2D	Self-reported (verified by medical records)	Chocolate (All-source)	Age, sex, ethnicity, BMI, smoking status, education, sleep duration, physical activity, DASH index, intakes of total energy, alcohol, coffee, soda, added sugar, red meat and dietary fiber	7.8	g/d<br>0<br>3<br>7<br>13	 1.00<br>0.97 (0.91, 1.03)<br>0.97 (0.91, 1.02)<br>0.90 (0.84, 0.97)
Matsumoto<br>et. al. (2015)	PHS<br>(USA)	PC	40-84	M	18235	1123	Validated FFQ	T2D	Self-reported (validated by medical records)	Chocolate (Pure)	Age, BMI, smoking, exercise, alcohol consumption, total caloric intake, intakes of whole grain, nuts, and red meat	9.2	g/d<br>0<br>1.9<br>4<br>10	 1.00<br>0.93 (0.79, 1.09)<br>0.86 (0.72, 1.04)<br>0.83 (0.69, 0.99)
Mink<br>et. al. (2007)	IWHS<br>(USA)	PC	55-69	W	34489	469	Validated FFQ	Death from stroke	Death certificates	Chocolate (Pure)	Age, energy intake, marital status, blood pressure, diabetes, BMI, waist-to-hip ration, physical activity, smoking, estrogen use	16	servings/wk<br>0<br>>0	 1.00<br>0.85 (0.70, 1.03)
						1329		Death from CHD					servings/wk<br>0<br>>0	 1.00<br>0.98 (0.88, 1.10)
Mostofsky<br>et. al. (2010)	SMC<br>(Sweden)	PC	49-83	W	31823	419	Validated FFQ	Heart failure	Medical records/ Death certificates	Chocolate (Pure)	Total energy intake, age, education, BMI, physical activity, cigarette smoking, living alone, postmenopausal hormone use, alcohol consumption, family history of MI before age 60, self-reported history of hypertension, self-reported history of high cholesterol	9	g/d<br>0<br>1.6<br>5.3<br>15.8<br>29.5	 1.00<br>0.74 (0.58, 0.95)<br>0.68 (0.50, 0.93)<br>1.09 (0.74, 1.62)<br>1.23 (0.73, 2.08)
Oba<br>et. al. (2010)	TS<br>(Japan)	PC	35-70	M	5897	278	Validated FFQ	T2D	Self-reported	Chocolate (All-source)	Age, smoking status, BMI, physical activity, length of education in years, alcohol consumption, total energy intake, fat intake, women’s menopausal	10	g/d<br>0<br>0.7<br>1.7	 1.00<br>0.84 (0.65, 1.09)<br>0.65 (0.43, 0.97)

				W	7643	175					status		g/d<br>0<br>0.7<br>1.9	 1.00<br>0.70 (0.50, 0.98) /<br>0.73 (0.48, 1.13)
Paganini-Hill et. al. (2007)	LWCS (USA)	PC	44-101	M&W	13624	11386	Questionnair e	All-cause mortality	Death certificates	Chocolate (All-source)	Age, sex, smoking, exercise, BMI, alcohol intake, history of hypertension, angina, heart attack, stroke, diabetes, rheumatoid arthritis and cancer	23	Frequency<br>None<br>Few times/y to few times/m<br>Few times/wk to daily	 1.00<br>0.94 (0.90, 0.98)<br><br>0.98 (0.93, 1.04)
Petrone et. al. (2014)	PHS (USA)	PC	50-98	M	20278	876	Validated FFQ	Heart failure	Self-reported (validated by medical records)	Chocolate (Pure)	Age, BMI, alcohol, smoking exercise, caloric intake, prevalent atrial fibrillation	9.3	g/d<br>0<br>1.9<br>4<br>12.1<br>24.2	 1.00<br>0.86 (0.72, 1.03)<br>0.80 (0.66, 0.98)<br>0.92 (0.74, 1.13)<br>0.82 (0.63, 1.07)
Singh et. al. (2013)	IWHS (USA)	PC	55-69	W	35216	1298	Validated FFQ	Colorectal cancer	Medical Records/ Pathology report	Chocolate (Pure)	NA	34	Frequency<br>Non-consumers<br>>1 times/wk	 1.00<br>1.14 (0.97, 1.33)
Steinhaus et. al. (2016)	CSM (Sweden)	PC	45-79	M	31917	2157	Validated FFQ	Heart failure	Medical records/ Death certificates	Chocolate (Pure)	Age, total energy intake, DASH diet component score, education, BMI, physical activity, cigarette smoking, alcohol consumption, family history of MI, self-reported history of hypertension and high cholesterol	14	g/d<br>0<br>3.4<br>7.3<br>21.9<br>46.1	 1.00<br>0.88 (0.78, 0.99)<br>0.83 (0.72, 0.94)<br>0.82 (0.68, 0.99)<br>1.10 (0.84, 1.45)

Study or Subgroup	Weight	Risk Ratio IV, Random, 95% CI
Janszky 2009 SHEEP	1.1%	0.94 [0.58, 1.53]
Paganini-Hill 2007 LWCS	98.9%	0.98 [0.93, 1.03]
**Total (95% CI)**	**100.0%**	**0.98 [0.93, 1.03]**

Heterogeneity: $Tau^2 = 0.00$; $Chi^2 = 0.03$, df = 1 (P = 0.87); $I^2 = 0\%$
Test for overall effect: Z = 0.78 (P = 0.44)

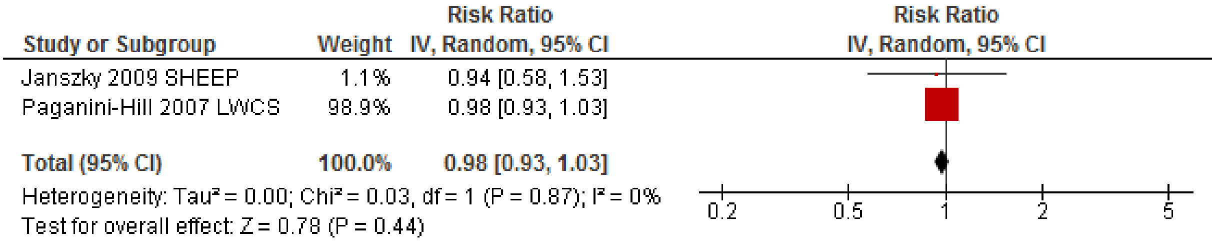

ESM Fig 2: Summary of relative risk of all-cause mortality for high versus low chocolate intake.

Study or Subgroup	Weight	Risk Ratio IV, Random, 95% CI
Buijsse 2010 EPIC-Potsdam	2.8%	0.73 [0.47, 1.15]
Greenberg 2018 WHI	16.9%	1.03 [0.92, 1.15]
Ho 2018 MVP	17.6%	0.89 [0.80, 0.99]
Hu 2000 NHS	10.7%	1.11 [0.92, 1.34]
Janszky 2009 SHEEP	2.7%	0.86 [0.54, 1.37]
Kwok 2015 EPIC-Norfolk	12.4%	0.82 [0.70, 0.97]
Larsson 2016 SMC + COSM	16.0%	0.87 [0.77, 0.98]
Lewis 2010 COOW	4.1%	0.65 [0.45, 0.94]
Mink 2007 IWHS	16.9%	0.98 [0.88, 1.10]
**Total (95% CI)**	**100.0%**	**0.92 [0.85, 1.00]**

Heterogeneity: $Tau^2 = 0.01$; $Chi^2 = 16.35$, df = 8 (P = 0.04); $I^2 = 51\%$
Test for overall effect: Z = 2.07 (P = 0.04)

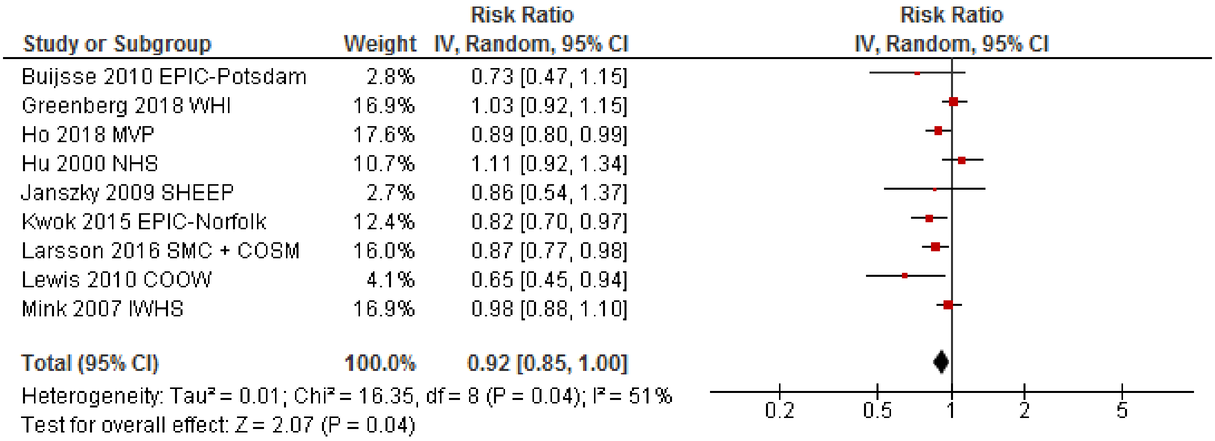

ESM Fig 3: Summary of relative risk of CHD for high versus low chocolate intake.

Study or Subgroup	Weight	Risk Ratio IV, Random, 95% CI
Janszky 2009 SHEEP	14.4%	0.78 [0.52, 1.16]
Kwok 2016 EPIC-Norfolk	25.6%	0.87 [0.71, 1.06]
Lewis 2010 COOW	7.8%	0.41 [0.22, 0.76]
Mostofsky 2010 SMC	10.0%	1.23 [0.73, 2.08]
Petrone 2014 PHS	21.4%	0.82 [0.63, 1.07]
Steinhaus 2017 COSM	20.8%	1.10 [0.84, 1.45]
**Total (95% CI)**	**100.0%**	**0.87 [0.71, 1.06]**

Heterogeneity: $Tau^2 = 0.03$; $Chi^2 = 10.54$, df = 5 (P = 0.06); $I^2 = 53\%$
Test for overall effect: Z = 1.42 (P = 0.16)

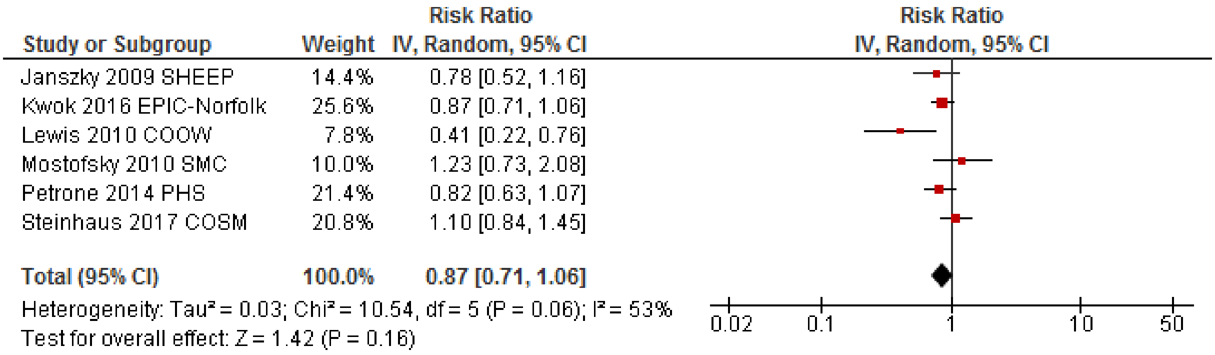

ESM Fig 4: Summary of relative risk of heart failure for high versus low chocolate intake.

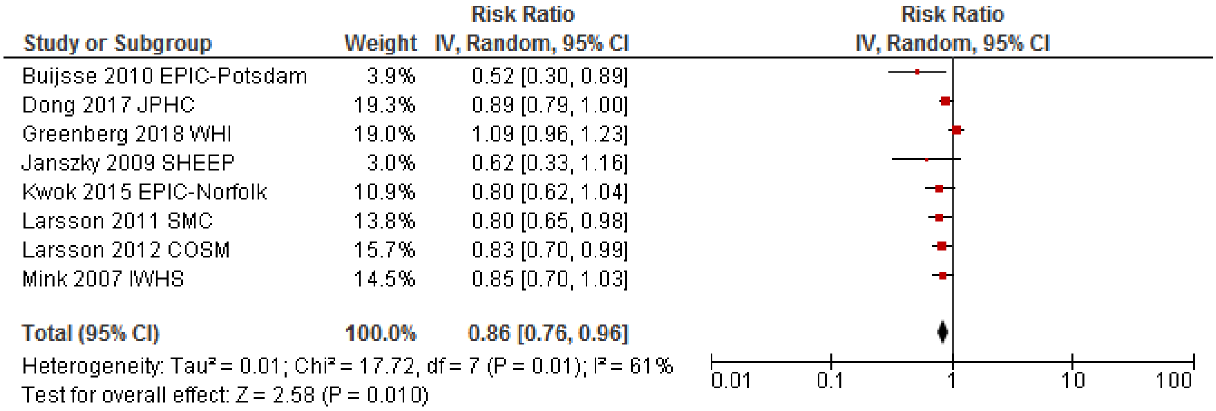

ESM Fig 5: Summary of relative risk of stroke for high versus low chocolate intake.

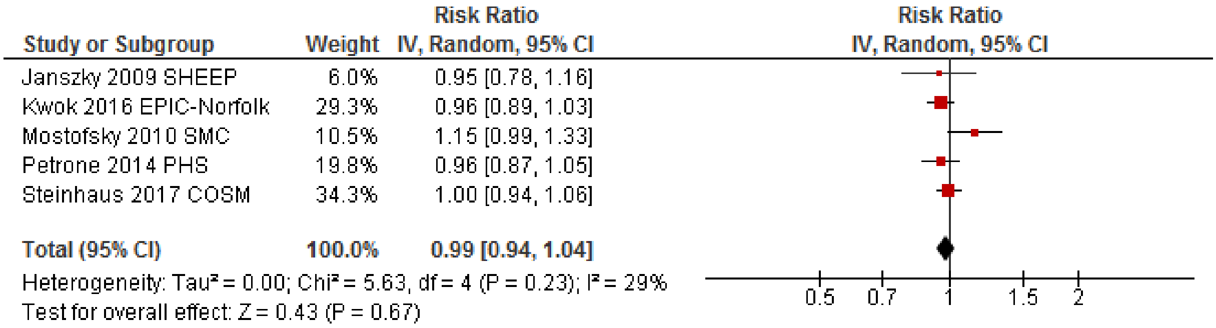

ESM Fig 6: Summary of relative risk of heart failure for each 10 g/d increase in chocolate intake.

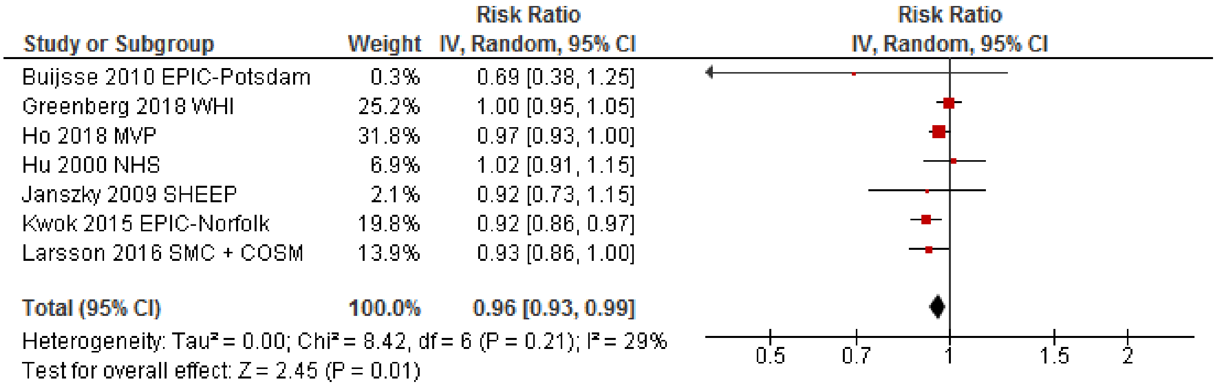

ESM Fig 7: Summary of relative risk of CHD for each 10 g/d increase in chocolate intake.

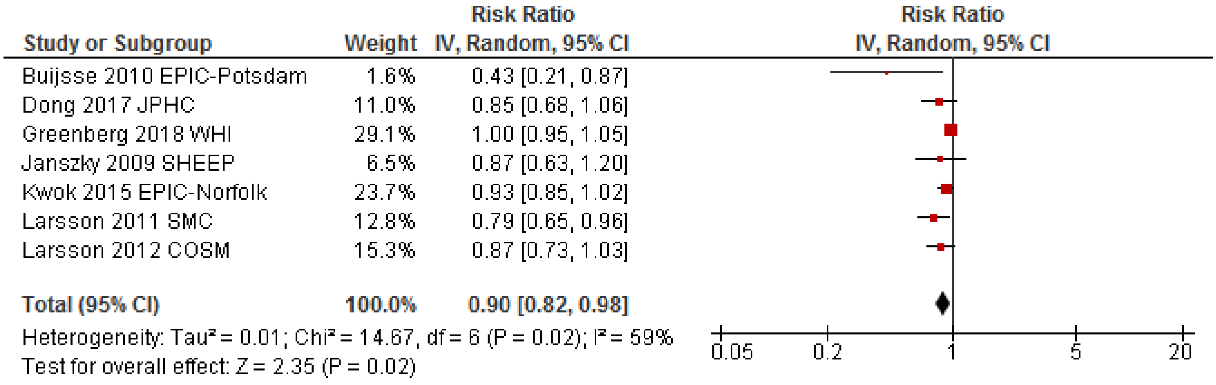

ESM Fig 8: Summary of relative risk of stroke for each 10 g/d increase in chocolate intake.

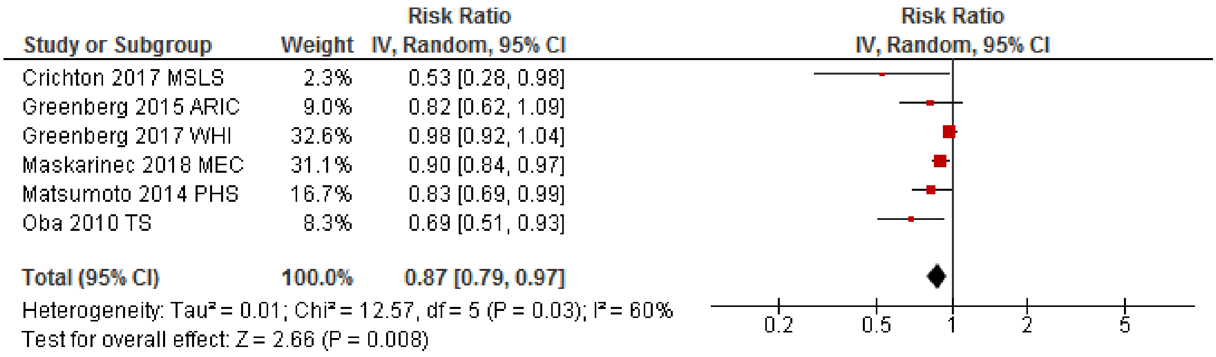

ESM Fig 9: Summary of relative risk of T2D for high versus low chocolate intake.

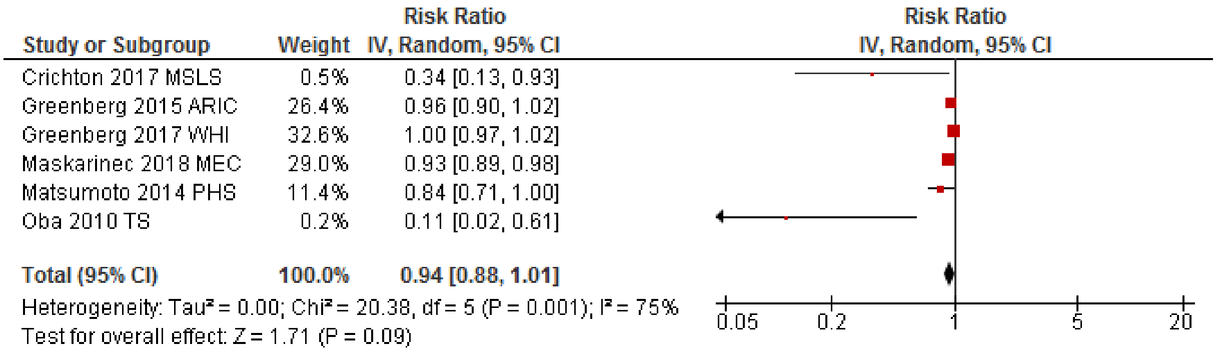

ESM Fig 10: Summary of relative risk of T2D for each 10 g/d increase in chocolate intake.

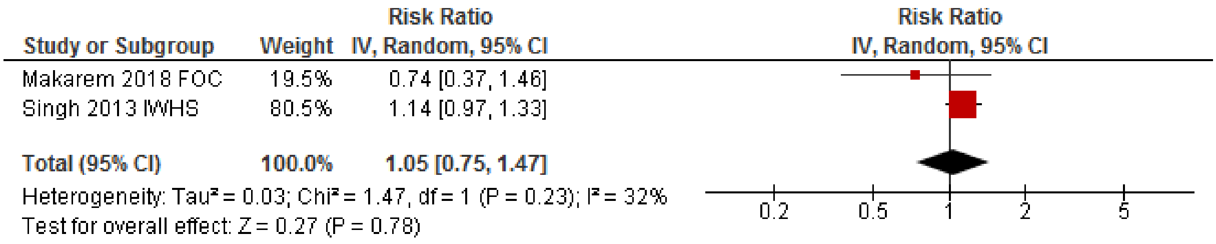

ESM Fig 11: Summary of relative risk of colorectal cancer for high versus low chocolate intake.

Study or Subgroup	Weight	Risk Ratio IV, Random, 95% CI
Alonso 2005 SUN	1.9%	1.10 [0.70, 1.74]
Lajous 2016 E3N	98.1%	0.97 [0.91, 1.03]
**Total (95% CI)**	**100.0%**	**0.97 [0.91, 1.04]**

Heterogeneity: $Tau^2 = 0.00$; $Chi^2 = 0.28$, $df = 1$ ($P = 0.59$); $I^2 = 0\%$
Test for overall effect: $Z = 0.87$ ($P = 0.38$)

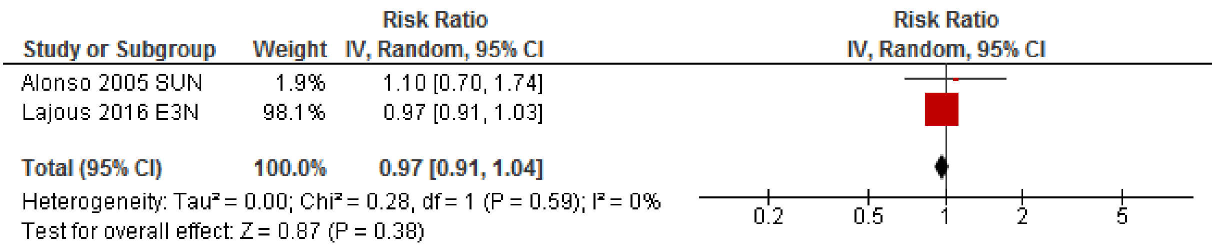

ESM Fig 12: Summary of relative risk of hypertension for high versus low chocolate intake.

ESM Table 2: Dose-response meta-analysis for each daily 10 gram increase in chocolate intake and risk of CHD, stratified by sex, follow-up, geographic location, and number of cases, dietary assessment, type of chocolate, and energy adjustment.

CHD	No of studies	RR	95% CI	I^2 (%)	p-value for subgroup difference
Dose-response	8	0.96	0.93, 0.99	29	
Sex					
Men and women	6	0.95	0.92, 0.97	0	
Women	2	1.00	0.96, 1.05	0	
Follow-up					
≥10 years	5	0.96	0.91, 1.01	57	0.93
<10 years	3	0.96	0.93, 1.00	0	
Geographic location					
Europe	5	0.92	0.88, 0.96	0	0.02
America	3	0.98	0.95, 1.01	0	
Number of cases					
≥1000	5	0.96	0.93, 0.99	50	0.65
<1000	3	0.98	0.88, 1.10	7	
Dietary assessment*					
not validated	1	0.92	0.73, 1.15	NA	0.72
validated	6	0.96	0.91, 1.01	51	
Type of chocolate					
Pure	5	0.96	0.93, 0.99	0	0.83
Combined	3	0.95	0.88, 1.04	68	
Energy adjusted					
Yes	6	0.96	0.91, 1.01	51	0.84
No	2	0.96	0.93, 1.00	0	

*No information for Ho et al. 2018 available

ESM Table 3: Dose-response meta-analysis for each daily 10 gram increase in chocolate intake and risk of stroke, stratified by sex, follow-up, geographic location, and number of cases, dietary assessment, type of chocolate, and energy adjustment.

Stroke	No of studies	RR	95% CI	I^2 (%)	p-value for subgroup difference
Dose-response	7	0.90	0.82, 0.98	59	
Sex					
Men and women	4	0.87	0.75, 1.01	39	
Men	1	0.87	0.73, 1.03	NA	0.77
Women	2	0.91	0.72, 1.14	81	
Follow-up					
≥10 years	5	0.92	0.84, 1.00	57	0.34
<10 years	2	0.66	0.33, 1.30	69	
Geographic location					
Europe	5	0.86	0.77, 0.96	37	0.03
America	1	1.00	0.95, 1.05	NA	
Asia	1	0.85	0.68, 1.06	NA	
Number of cases					
≥1000	4	0.90	0.79, 1.02	65	0.63
<1000	3	0.83	0.64, 1.09	56	
Dietary assessment					
not validated	1	0.87	0.63, 1.20	NA	0.88
validated	6	0.89	0.81, 0.99	65	
Type of chocolate					
Pure	4	0.84	0.76, 0.93	0	0.17
Combined	3	0.94	0.83, 1.07	71	
Energy adjusted					
Yes	6	0.89	0.81, 0.99	65	0.88
No	1	0.87	0.63, 1.20	NA	

ESM Table 4: Dose-response meta-analysis for each daily 10 gram increase in chocolate intake and risk of T2D, stratified by sex, follow-up, geographic location, and number of cases, dietary assessment, type of chocolate, and energy adjustment.

T2D	No of studies	RR	95% CI	I^2 (%)	p-value for subgroup difference
Dose-response	6	0.94	0.88, 1.01	75	
Sex					
Men and women	4	0.92	0.82, 1.04	71	
Men	1	0.84	0.71, 1.00	NA	0.05
Women	1	1.00	0.97, 1.02	NA	
Follow-up					
≥10 years	4	0.96	0.86, 1.07	76	0.50
<10 years	2	0.91	0.84, 0.99	26	
Geographic location					
Europe	5	0.95	0.89, 1.01	72	0.01
Asia	1	0.11	0.02, 0.61	NA	
Number of cases					
≥1000	3	0.95	0.88, 1.02	78	0.16
<1000	3	0.42	0.13, 1.33	80	
Dietary assessment					
not validated	2	0.65	0.24, 1.72	75	0.46
validated	4	0.94	0.86, 1.03	81	
Type of chocolate					
Pure	2	0.62	0.27, 1.42	67	0.30
Combined	4	0.96	0.88, 1.01	75	
Energy adjusted					
Yes	5	0.95	0.89, 1.01	75	0.05
No	1	0.34	0.13, 0.93		

ESM Table 5: Highest vs. lowest intake category meta-analysis, or linear dose-response meta-analysis NutriGrade judgements between chocolate intake and the risk of all-cause mortality, T2D, CHD, stroke, heart failure, colorectal cancer, and hypertension.

**Outcome**	**Comparison**	**No of studies**	**RR**	**95% CI**	**I^2 (%)**	**NutriGrade grading**
All-cause mortality	Highest vs. lowest	2	0.98	0.93, 1.03	0	Very low
CHD	Per 10 g/d	8	0.96	0.93, 0.99	29	Low
Stroke	Per 10 g/d	7	0.90	0.82, 0.98	59	Low
Heart Failure	Per 10 g/d	5	0.99	0.94, 1.04	29	Very low
T2D	Per 10 g/d	6	0.94	0.88, 1.01	75	Very low
Colorectal cancer	Highest vs. lowest	2	1.05	0.75, 1.47	32	Very low
Hypertension	Highest vs. lowest	2	0.97	0.91, 1.04	0	Very low

ESM Ref

1. Buijsse B, Feskens EJ, Kok FJ, Kromhout D (2006) Cocoa intake, blood pressure, and cardiovascular mortality: the Zutphen Elderly Study. Arch Intern Med 166 (4):411-417. doi:10.1001/archinte.166.4.411

2. Arts IC, Hollman PC, Feskens EJ, Bueno de Mesquita HB, Kromhout D (2001) Catechin intake might explain the inverse relation between tea consumption and ischemic heart disease: the Zutphen Elderly Study. Am J Clin Nutr 74 (2):227-232. doi:10.1093/ajcn/74.2.227

3. Diaz-Lopez A, Bullo M, Martinez-Gonzalez MA, Corella D, Estruch R, Fito M, Gomez-Gracia E, Fiol M, Garcia de la Corte FJ, Ros E, Babio N, Serra-Majem L, Pinto X, Munoz MA, Frances F, Buil-Cosiales P, Salas-Salvado J (2016) Dairy product consumption and risk of type 2 diabetes in an elderly Spanish Mediterranean population at high cardiovascular risk. Eur J Nutr 55 (1):349-360. doi:10.1007/s00394-015-0855-8

4. Dower JI, Geleijnse JM, Hollman P, Soedamah-Muthu SS, Kromhout D (2016) Dietary epicatechin intake and 25-y risk of cardiovascular mortality: the Zutphen Elderly Study. Am J Clin Nutr 104 (1):58-64. doi:10.3945/ajcn.115.128819

5. Ivey KL, Hodgson JM, Croft KD, Lewis JR, Prince RL (2015) Flavonoid intake and all-cause mortality. Am J Clin Nutr 101 (5):1012-1020. doi:10.3945/ajcn.113.073106

6. Ponzo V, Goitre I, Fadda M, Gambino R, De Francesco A, Soldati L, Gentile L, Magistroni P, Cassader M, Bo S (2015) Dietary flavonoid intake and cardiovascular risk: a population-based cohort study. Journal of translational medicine 13:218. doi:10.1186/s12967-015-0573-2

7. Semba RD, Ferrucci L, Bartali B, Urpi-Sarda M, Zamora-Ros R, Sun K, Cherubini A, Bandinelli S, Andres-Lacueva C (2014) Resveratrol levels and all-cause mortality in older

community-dwelling adults. JAMA Intern Med 174 (7):1077-1084. doi:10.1001/jamainternmed.2014.1582

8. Vogiatzoglou A, Mulligan AA, Bhaniani A, Lentjes MAH, McTaggart A, Luben RN, Heiss C, Kelm M, Merx MW, Spencer JPE, Schroeter H, Khaw KT, Kuhnle GGC (2015) Associations between flavan-3-ol intake and CVD risk in the Norfolk cohort of the European Prospective Investigation into Cancer (EPIC-Norfolk). Free Radic Biol Med 84:1-10. doi:10.1016/j.freeradbiomed.2015.03.005

9. Balboa-Castillo T, Lopez-Garcia E, Leon-Munoz LM, Perez-Tasigchana RF, Banegas JR, Rodriguez-Artalejo F, Guallar-Castillon P (2015) Chocolate and health-related quality of life: a prospective study. PLoS One 10 (4):e0123161. doi:10.1371/journal.pone.0123161

10. Crichton GE, Elias MF, Alkerwi A, Stranges S, Abhayaratna WP (2016) Relation of Habitual Chocolate Consumption to Arterial Stiffness in a Community-Based Sample: Preliminary Findings. Pulse (Basel, Switzerland) 4 (1):28-37. doi:10.1159/000445876

11. Frost L, Vestergaard P (2005) Caffeine and risk of atrial fibrillation or flutter: the Danish Diet, Cancer, and Health Study. Am J Clin Nutr 81 (3):578-582. doi:10.1093/ajcn/81.3.578

12. Jhee JH, Nam KH, An SY, Cha MU, Lee M, Park S, Kim H, Yun HR, Kee YK, Park JT, Chang TI, Kang EW, Yoo TH, Kang SW, Han SH (2018) Effects of Coffee Intake on Incident Chronic Kidney Disease: A Community-Based Prospective Cohort Study. Am J Med 131 (12):1482-1490.e1483. doi:10.1016/j.amjmed.2018.05.021

13. Khawaja O, Petrone AB, Kanjwal Y, Gaziano JM, Djousse L (2015) Chocolate Consumption and Risk of Atrial Fibrillation (from the Physicians' Health Study). Am J Cardiol 116 (4):563-566. doi:10.1016/j.amjcard.2015.05.009

14. Mostofsky E, Berg Johansen M, Tjonneland A, Chahal HS, Mittleman MA, Overvad K (2017) Chocolate intake and risk of clinically apparent atrial fibrillation: the Danish Diet, Cancer, and Health Study. Heart 103 (15):1163-1167. doi:10.1136/heartjnl-2016-310357

15. Saftlas AF, Triche EW, Beydoun H, Bracken MB (2010) Does chocolate intake during pregnancy reduce the risks of preeclampsia and gestational hypertension? Annals of epidemiology 20 (8):584-591. doi:10.1016/j.annepidem.2010.05.010

16. Stellman SD, Garfinkel L (1988) Patterns of artificial sweetener use and weight change in an American Cancer Society prospective study. Appetite 11 Suppl 1:85-91

17. Strandberg TE, Strandberg AY, Pitkala K, Salomaa VV, Tilvis RS, Miettinen TA (2008) Chocolate, well-being and health among elderly men. Eur J Clin Nutr 62 (2):247-253. doi:10.1038/sj.ejcn.1602707

18. Triche EW, Grosso LM, Belanger K, Darefsky AS, Benowitz NL, Bracken MB (2008) Chocolate consumption in pregnancy and reduced likelihood of preeclampsia. Epidemiology (Cambridge, Mass) 19 (3):459-464. doi:10.1097/EDE.0b013e31816a1d17

19. Uccella S, Mariani A, Wang AH, Vierkant RA, Cliby WA, Robien K, Anderson KE, Cerhan JR (2013) Intake of coffee, caffeine and other methylxanthines and risk of Type I vs Type II endometrial cancer. Br J Cancer 109 (7):1908-1913. doi:10.1038/bjc.2013.540

20. di Giuseppe R, Di Castelnuovo A, Centritto F, Zito F, De Curtis A, Costanzo S, Vohnout B, Sieri S, Krogh V, Donati MB, de Gaetano G, Iacoviello L (2008) Regular consumption of dark chocolate is associated with low serum concentrations of C-reactive protein in a healthy Italian population. J Nutr 138 (10):1939-1945. doi:10.1093/jn/138.10.1939
